# Guideline “diagnosis and non interventional therapy of neuropathic pain” of the German Society of Neurology (deutsche Gesellschaft für Neurologie)

**DOI:** 10.1186/s42466-020-00063-3

**Published:** 2020-06-10

**Authors:** Tanja Schlereth

**Affiliations:** DKD Helios Hospital Wiesbaden, Aukammallee 33, 65191 Wiesbaden, Germany

**Keywords:** Neuropathic pain, Guideline, Diagnosis, Therapy, Pregabalin, Gabapentin, Duloxetine, Amitriptyline, Capsaicin, Lidocaine

## Abstract

2019 the DGN (Deutsche Gesellschaft für Neurology) published a new guideline on the diagnosis and non-interventional therapy of neuropathic pain of any etiology excluding trigeminal neuralgia and CRPS (complex regional pain syndrome). Neuropathic pain occurs after lesion or damage of the somatosensory system. Besides clinical examination several diagnostic procedures are recommended to assess the function of nociceptive A-delta and C-Fibers (skin biopsy, quantitative sensory testing, Laser-evoked potentials, Pain-evoked potentials, corneal confocal microscopy, axon reflex testing). First line treatment in neuropathic pain is pregabalin, gabapentin, duloxetine and amitriptyline. Second choice drugs are topical capsaicin and lidocaine, which can also be considered as primary treatment in focal neuropathic pain. Opioids are considered as third choice treatment. Botulinum toxin can be considered as a third choice drug for focal limited pain in specialized centers only. Carbamazepine and oxcarbazepine cannot be generally  recommended, but might be helpful in single cases. In Germany, cannabinoids can be prescribed, but only after approval of reimbursement. However, the use is not recommended, and can only be considered as off-label therapy within a multimodal therapy concept.

## What’s new?

- In 2017, a new law (“Cannabis Act”) came into force, which made cannabinoids (cannabis flowers, cannabis extracts, dronabinol, nabilone, nabiximols) reimbursable in Germany upon application to health insurance and they can be prescribed via narcotics (BTM) prescription. However, it is still an off-label use, as none of these substances is approved for the indication “pain”.
- In addition to neuropathic and nociceptive pain, the IASP has also defined the entity of “nociplastic” pain (see below)
- Mutations of the voltage-gated sodium channels (Nav1.7, Nav1.8 und Nav1.9) and TRPA1 have been identified as possible causes of idiopathic small-fiber neuropathy
- Corneal confocal microscopy is an non-invasive diagnostic tool for the diagnosis of small-fiber neuropathies
- In 2015, the NeuPSIG (Neuropathic Pain Special Interest Group of the IASP = International Association for the Study of Pain) prepared current therapy recommendations for the treatment of neuropathic pain following a systematic literature research and meta-analysis, which were taken into account in this guideline.

## The most important recommendations

- Neuropathic pain occurs as a result of damage or lesion of the somatosensory system.
- The diagnosis neuropathic pain is based on the typical symptoms and findings of neuropathic pain, in particular the combination of minus symptoms (sensory deficits such as hypaesthesia, hypalgesia) and plus symptoms (burning pain, especially at rest, shooting pain, allodynia, hyperalgesia).
- The damage or lesion of the somatosensory system must be demonstrated by neurological examination and confirmed by means of instrument-based diagnostics.
- Skin biopsy is still- with limitations- considered as the gold standard in the diagnosis of small-fiber neuropathies. Established non-invasive methods are quantitative sensory testing (QST) and laser-evoked potentials, the latter being available only in specialized centers. Pain-evoked potentials, corneal confocal microscopy, and axon reflex testing are also methods for detection of C- or A- delta fiber damage, but are usually only available in specialized centers and validated standard values do not exist for all tests.
- Questionnaires can be uses as a screening tool or to assess the severity of the neuropathy
- The possibility of curative or causal therapy (e.g. neurolysis in nerve entrapment syndromes, optimal diabetes control in diabetic neuropathy) should be explored.
- Each patient requires an individual dosage depending on the efficacy and side effects.
- First choice for pharmacological treatment are anticonvulsants with effects on neuronal calcium channels (gapapentin/pregabalin) as well as tri- and tetracyclic antidepressants and the selective serotonin−/norepinephrine reuptake inhibitor duloxetine (However, the latter is in Germany only approved for the treatment of diabetic neuropathy).
- For focal nerve lesions topical lidocaine 5%- and capsaicin 8% patches are effective and should be preferred due to fewer side effects.
- Opioids are effective, but attention should be paid to their side effects and potential for dependence. High-potency opioids as well as the low potency opioid tramadol, which additionally acts on the endogenous descending pain inhibition, can be recommended as third choice drugs.
- Carbamazepine and oxcarbazepine cannot be genenerally recommended for neuropathic pain due to limited evidence and frequent side effects, but might be considered on a case-by-case basis. However, for trigeminal neuralgia (see seperate guideline) carbamazepine is still the drug of first choice.
- Lamotrigine cannot be generally recommended due to lack of data, although there is evidence from smaller individual studies of an effect on HIV neuropathy and central pain.
- Combination of medication may be useful, as they can reduce the individual doses and enable synergistic effects.

## Introduction

Neuropathic pain occurs after a lesion or damage of the somatosensory system (definition of the Neuropathic Pain Special Interest Group (NeuPSIG) of the International Association for the Study of Pain (IASP) [1]. In contrast, nociceptive pain is caused by activation of nociceptors, whereas neuronal structures are basically intact (e.g. pain in osteoarthritis). Nociplastic pain (new definition of the IASP 2017) is pain arisen from altered nociception despite no clear evidence of actual or threatened tissue damage causing the activation of peripheral nociceptors or evidence for disease or lesion of the somatosensory system causing pain. The prevalence of neuropathic pain is 6.9–10% [2]. The treatment of neuropathic pain is different from the treatment of other chronic pain in which the somatosensory nervous system is not damaged (nociceptive and / or nociplastic pain.

## Methods of guideline development

AWMF registry number 030/114.

Update: Mai 2019.

Valid until April 2024.

Edited by the German Society of Neurology (Deutsche Gesellschaft für Neurologie).

### Pathophysiology

Pathophysiologically, the development of pathological spontaneous activity in damaged and intact nociceptive afferents as a result of biochemical, physiological, morphological, and partly genetic changes has been shown for many neuropathic pains. The lesion induces plastic changes in the peripheral and central nervous system, with an imbalance between excitatory and inhibitory mechanisms and disturbed descending inhibitory mechanisms [3, 4].

### Grading of neuropathic pain

A distinction is made between possible, probable, and definite neuropathic pain [1, 5, 6]. In the absence of any criteria, the diagnosis is “unlikely”. In detail, the criteria are as follows:

1. The medical history indicates a relevant lesion or disease of the peripheral or central somatosensory system.
2. Pain is localized in a neuro-anatomically plausible area.
3. There is at least one pathological sensory finding within the neuro-anatomically plausible area of pain propagation.
4. A relevant lesion or disease of the peripheral or central somatosensory system can be detected by at least one examination.

**Recommendation:** The grading of neuropathic pain **should be** used in the diagnosis of neuropathic pain of any etiology. It is a helpful tool for identifying and classifying neuropathic pain.

### Peripheral and/or central neuropathic pain

A distinction has to be made between peripheral and central neuropathic pain, since the latter is often more difficult to treat.

### Neuropathic pain – nociceptive pain – mixed pain

The presence of a neuropathic pain component does not exclude an additional nociceptive pain component in the same patient (e.g. foot ulcer pain and additional painful diabetic neuropathy). In some cases - so called mixed pain syndromes- a clear distinction is not always possible.

#### Peripheral focal or multifocal painful neuropathies

- acute herpes zoster, postherpetic neuralgia
- post-mastectomy-pain, post-thoracotomy-pain, scar pain
- phantom limb pain, pain after nerve injury
- posttraumatic neuropathy
- trigeminal neuralgia, glossopharyngeal neuralgia, occipital neuralgia
- acute and chronic radicular pain, post-discectomy pain syndrome,
- nerve entrapment syndroms
- diabetic mononeuropathy
- Morton’s neuroma
- Ischemic neuropathy
- Bannwarth’s syndrome (Borrelia infection)
- Idiopathic plexus neuritis, plexus lesion after radiation or tumor
- Special situation: complex regional pain syndrome (CRPS; see DGN guideline “CRPS”)

#### Peripheral painful polyneuropathies (see also DGN guideline “polyneuropathy”)

- Metabolic
  - diabetes, hypothyroidism, vitamin deficiency (especially vitamin B12)
- Medication
  - Antiretroviral therapy, chemotherapy (cisplatin, oxaliplatin, Taxanes, thiouracil, vincristine), disulfiram, antibiotics (ethambutol, isoniazid, nitrofurantoin, chloramphenicol, metronidazole), thalidomide, gold
- Toxins
  - alcohol, acrylamid, arsenic, clioquinol, dinitrophenol, ethylenoxide, pentachlorphenol, thallium
- Hereditary
  - Amyloidosis, Fabry’s diesase, Charcot-Marie-Tooth disease, hereditary sensory autonomic neuropathies (HSAN), primary erythromelalgia (e.g. gene mutation of the voltage gated sodium channel NaV1.7)
- Malignancies
  - paraneoplastic (especially bronchial carcinoma), multiple myeloma
- Infections, autoimmune diseases
  - acute inflammatory polyradiculoneuropathy (Guillain-Barré syndrome), chronic inflammatory demyelinating Polyradikuloneuropathy (CIDP), vasculitic neuropathy
  - HIV-neuropathy, leprosy
- Polyneuropathies of other etiology

#### Causes of central neuropathic pain

- vascular
  - ischemia (e.g. insula, thalamus, brain stem), hemorrage, vascular malformations
- inflammation
  - multiple skleroses, abscess, myelitis
- trauma
- malignancies
- syringomyelia

**“mixed pain”-syndrome**

- chronic back pain
- cancer pain (with infiltration of neuronal structures)

## Diagnostics

### Medical history

Assessment of medical history serves to distinguish pain syndromes (nociceptive or nociplastic vs. neuropathic). It should provide information about relevant lesions or diseases of the peripheral or central somatosensory system. Information about impairment, previous treatment, and pain- relevant comorbidities such as anxiety, depression, or sleep disorders is also essential.

### Clinical symptoms

Due to the lesion of afferent nerve fibers, many patients report numbness. These “negative” sensory symptoms are unpleasant and can lead to disability, but are usually not painful and cannot be influenced by medication. Characteristic symptoms of neuropathic pain such as evoked-pain and pain hypersensitivity (hyperalgesia, and allodynia) are called “positive” sensory symptoms and usually require specific therapy. Neuropathic pain is usually spontaneous pain (without external stimulation). Pain quality is usually burning. In contrast to nociceptive pain, symptoms are typically not dependent on physical exertion or movement. Frequently, spontaneous pain attacks of shooting, lancinating, stabbing quality occur. In polyneuropathies, the pain can manifest as pressure or tightness in the limb and paresthesias and dysesthesias are common. Some patients have itching, muscle cramps or restlessness similar to the classical restless legs syndrome. External stimuli can elicit evoked pain. In the case of allodynia, pain is evoked by non-noxious stimulation (touch, non painful warm or cold). Hyperalgesia is present when a primarily slightly painful stimulus triggers a more intense pain compared to healthy subjects [1, 7]. Deafferentiation pain occurs after disruption of large nerve structures (e.g. after amputation) or spinal cord lesions [3].

### Measurement of pain intensity and localization

Pain intensity can be quantified using an 11-part numerical rating scale (NRS), on which pain intensity is classified on a scale from 0 (“no pain”) to 10 (“maximal imaginable pain”). An alternative is the visual analogue scale (with the endpoints “no pain” and “maximal imaginable pain” on a 10 cm horizontal line. In children, Likert scales consisting of 4–5 verbal descriptors or Icons can be used.

### Assessment of pathological findings

A thorough neurological examination should always be performed to detect pathological findings within the pain area and to identify the distribution pattern of symptoms, which would indicate a lesion of the somatosensory system. Sensory symptoms (touch, mechanical sensitivity, temperature sensation, proprioception, vibration sense), muscle strength and reflexes should be assessed.

### Quantitative sensory testing

The quantitative sensory testing (QST) is a standardized procedure to assess sensitivity of the skin and deeper structures (muscles, fascias). The German Research Network (Deutscher Forschungsverbund Neuropathischer Schmerz (DFNS)) recommends a combination of different tests to assess detection and pain thresholds of thermal, mechanical, sensory, and nociceptive parameters to assess the function of non-myelinated C-fibers and thin myelinated Aδ-fibers and their nerve tracts. Deviations from normal values can be determined by z-value transformation, which allows creating sensitivity profiles. Age and gender-specific reference data of the DFNS exist. QST can detect positive symptoms like allodynia, mechanical, heat, or cold hyperalgesia as well as negative symptoms (loss of mechanical or thermal sensation). The detection of negative symptoms is important for the diagnosis of neuropathic pain [6]. Negative symptoms can occur with nociceptive pain also, but are less pronounced, have no neuro-anatomically plausible pain distribution and are not reproducible [8]. Since active cooperation of the patient is required, under certain circumstances (e.g. lack of cooperation, cognitive or linguistic limitation) QST may not provide valid results.

There is a clear indication for QST in patients with suspected small-fiber neuropathy, with increased thermal detection thresholds, although sensitivity is significantly lower compared to skin biopsy. QST is currently recommended by NeuPSIG (Neuropathic Pain Special Interest Group of the International Association for the Study of Pain) and the EFNS (European Federation of Neurological Societies) as an additional laboratory test for the diagnosis of neuropathic pain [9–11].

**Recommendation:** Quantitative sensory testing **can be** used in the diagnosis of neuropathic pain of any etiology, especially if conventional electrophysiological methods do not show any abnormalities and/or there is a suspicion of an affection of small nerve fibers (small fiber neuropathy) or the associated central pathways. QST does not allow exact localization of the location of the nerve lesion or the differentiation between central and peripheral lesions or the etiological classification of the lesion.

### Skin biopsy

Skin punch biopsy is a minimally invasive procedure for obtaining a few millimeters of skin, in which intraepidermal C-fibers can be stained immunohistochemically. It is primarily used in the diagnosis of small-fiber neuropathies (SFN). A typical finding is the reduction of intraepidermal nerve fiber density (IENFD). A normal IENFD does not exclude neuropathic pain and small fiber neuropathy. A correlation between IENFD and pain intensity has only been reported in a few studies. There are no meta-analysis and no systematic reviews of skin biopsies for neuropathic pain. Skin biopsy is a procedure that can be used to confirm a somatosensory lesion in neuropathic pain. The NeuPSIG guidelines and European federation of neurological societies recommend the use of skin biopsy in the diagnosis of suspected small-fiber neuropathy [11, 12]. If performed in a standardized manner (with regard to collection site, method, staining technique, IENFD quantification) skin punch biopsy is an objective procedure for neuropathic pain syndromes with suspected small-fiber pathology.

**Recommendation:** Skin biopsy **can be** used in the diagnosis of neuropathic pain as well as small-fiber neuropathies, especially if other electrophysiological methods do not show any abnormalities and/or small-fiber pathology is suspected.

### Laser-evoked potentials (LEP)

Laser-evoked potentials can measure the function of the nociceptive system objectively. After stimulation of the skin (e.g. hand), potentials can be recorded on the scalp with EEG similar to somatosensory evoked potentials (SEP). In contrast to SEP, in which a nerve is electrically stimulated (especially thickly myelinated, rapidly conducting A-beta-fibers), the nociceptors in the epidermis are directly stimulated thermally and without contact by laser, which induces a specific stimulation of thin A-delta- or C-fibers. This enables functional testing of thin nerve fibers and the spinothalamic tract. A lesion of the somatosensory pain pathways leads to latency delays and/or amplitude reductions. Special techniques are used to differentiate the function of A-delta and C-fibers. In contrast to SEP, LEP can be derived from skin areas at the trunk or the trigeminal region. LEP amplitudes are an objective marker of damage to the nociceptive pathways (the smaller the amplitude, the greater/more relevant the damage), but less correlative of neuropathic pain intensity [13]. They are typically not enlarged, but reduced in pathological cases and thus particularly sensitive to negative symptoms. Most studies used LEP for the diagnosis of small-fiber neuropathies, spinal cord and brainstem lesions [14]. LEP are recommended by the European federation of neurological societies (EFNS [10];) and the IASP [11] for the diagnosis of neuropathic pain. However, this examination can only be carried out in a few specialized centers.

**Recommendation:** Laser-evoked potentials (LEP) **can be** used in the diagnosis of neuropathic pain of any etiology. However, they are not routinely used due to the high technical and time expenditure involved.

### Pain-related evoked potentials (PREP)

Pain-related evoked potentials (PREP) are derived by using concentric surface electrodes and stimulation with low electrical current intensities, which stimulate epidermal A-delta fibers. The generated potential can be derived at the Cz scalp electrode. To date, there are no meta-analyses and systematic reviews of the use of PREP in patients with neuropathic pain. It is not entirely clear whether PREP are derived from intraepidermal nerve endings of A-delta fibers or whether excitation of thickly myelinated dermal A-beta-fibers also occurs. In patients with neuropathic pain, PREP amplitudes were reduced compared to healthy individuals and correlated with pain severity [15]. In a study of patients with mixed neuropathy (small-fiber neuropathy and polyneuropathy), a reduction in PREP amplitudes was also shown to correlate with thermal perception thresholds and pain [16]. It is a simple, inexpensive and non-invasive procedure, which is, however, susceptible to interference and is currently only available at specialized centers. The current data is not sufficient to prove selective stimulation of A-delta-fibers in PREP.

**Recommendation:** PREP **can be** used in the diagnosis of neuropathic pain of any etiology.

### Corneal confocal microscopy

The in vivo corneal confocal microscopy (CCM) is a non-invasive, and rapid method for quantitative examination of the corneal nerve fibers of the subbasal plexus (between basal lamina and Bowman’s membrane), which, however, must be performed by trained examiners under standardized conditions. The small nerve fibers originate from the ophthalmic nerve as a branch of the trigeminal nerve and correspond to A-delta and C-fibers with low-threshold polymodal receptors for nociceptive, mechanical and cold stimuli. The most important parameters measured in CCM are corneal nerve fiber length (CNFL), nerve fiber density (CNFD), and number of nerve branches (CNBD [17];). While some studies have shown an albeit moderate correlation between the results of CCM and the extent of the neuropathy [18], the results on the correlation with intraepidermal nerve fiber density (IENFD) as the gold standard in the diagnosis of SFN are contradictory [18, 19].

**Recommendation:** CCM **can be** used in the diagnosis of neuropathic pain, especially if conventional electrophysiological methods do not show any abnormalities and/or there is a suspicion of an affection of small nerve fibers (SFN). It is important that CCM is performed by trained examiners and that ophthalmological abnormalities that lead to change in the corneal subbasal plexus are recorded and, if necessary, clarified (e.g. dry eye syndrome, contact lenses wearers, keratoconus, keratopathy, keratitis, ophthalmological surgery).

### Axon reflex tests

By determining the size of the axon reflex erythema, the function of afferent peripheral C-fibers (nociceptors) can be investigated. When peripheral C-fibers are activated, the action potentials spread throughout the axonal tree in the skin. In terminal nerve endings the action potentials trigger the release of the neuropeptide calcitonin gene-related peptide (CGRP), which causes a vasodilation in the skin and becomes visible as redness (neurogenic flare) [20, 21]. When C-fibers degenerate in the skin, the axon reflex erythema becomes smaller [22, 23]. The size of axon reflex erythema does not correlate with the severity of spontaneous pain in patients with neuropathic pain [24, 25]. Axon reflex erythema can be elicited chemically, for example with histamine [26] and acetylcholine [27], mechanically [28], by heat [29], or electrically. The extent of the skin reddening can be quantified by a laser-Doppler-Imager [30] or other methods such as laser speckle contrast analysis [31]. The measurement of the size of the axon reflex erythema is a functional, objective, and non-invasive method to assess afferent C-fiber function in humans, but is not clinically established and is only available in special centres for experimental purposes.

**Recommendations:** Axon reflex tests **can be** used in the diagnosis of neuropathic pain of any etiology.

### Questionnaires

There are various questionnaires that can be used to assess neuropathic pain symptoms qualitatively and quantitatively. The following are frequently used for the screening of neuropathic pain: painDETECT [32], DN4: Douleur Neuropathique en 4 Questions [33], LANSS: Leeds Assessment of Neuropathic Symptoms and Signs [34]. The extent of the neuropathic component of chronic pain syndromes can be assessed with the Neuropathic Pain Symptom Inventory (NPSI) [35] and the NPS: Neuropathic Pain Scale [36]. Some of the questionnaires are filled out by the patient (painDETECT, NPSI, NPS), while others contain, in addition to the patient survey, some points that have to be filled out by the examiner after a short clinical examination (DN4, LANSS). In general, it is recommended to use scales to record pain characteristics, which are typical of neuropathic pain (positive and negative symptoms), to measure the intensity of pain, and to include a full body drawing to estimate the localization and spreading of the symptoms.

Since psychological and social factors can modulate and maintain the experience and behavior of the patient, it is also useful to record these factors. The pain questionnaire of the German Pain Society (Deutsche Schmerzgesellschaft; www.dgss.org/deutscher-schmerzfragebogen/), for example, is suitable for this. It includes questions about the emotional and functional impairment caused by the pain, a screening for anxiety or depression as a possible co-morbidity, as well as questions about quality of life and social situation, and allows assessment of the degree of chronification.

**Recommendation:** Standardized questionnaires for pain characterization **should be used** in the diagnosis of neuropathic pain of any etiology. They can provide a good overview of subjective pain perception and the psychosocial component of pain as a supplement to clinical diagnostics, but without clinical examination they are not suitable as the sole means of diagnosing neuropathic pain.

### Diagnosis of the underlying lesion or disease of the somatosensory system

The previously mentioned methods have focused on the detection of damage to the nociceptive system, in particular the afferent C- and A-delta-fibers, as the cause of neuropathic pain. The basis for further diagnostics is a complete neurological examination. Further apparatus- based examinations (somatosensory evoked potentials, neurography, imaging methods such as MRI or CT), laboratory or cerebral spinal fluid examinations should be performed depending on the patient’s medical history and clinical findings. For details we refer to the guidelines for the corresponding diseases.

## Therapy

### General recommendations for drug therapy

Realistic therapy goals for neuropathic pain are:

- Pain reduction by ≥30%
- Improvement of sleep quality
- Improvement of quality of life
- Preservation of social activity and relationships
- Maintaining the ability to work
- Improved functionality

Neuropathic pain is a therapeutic challenge, since often freedom from pain cannot be achieved and, with all drug options, some patients respond poorly or suffer from intolerable side effects. The therapeutic goals must therefore be discussed realistically. Before starting therapy, potential side effects should be clarified to improve compliance. Patients should also be informed that the effect starts only after reaching an effective dose and with a time delay to avoid early discontinuation of potentially effective preparations. It may be useful and more effective to combine several drugs, as this may result in synergistic pain-relieving effects and the individual doses may remain lower [37].

The approval status of the individual substances must be taken into account, since some substances might be used off-label. To use them, the following off-label use criteria must be met.

1. Established effect
2. Favorable benefit-to-risk profile
3. Missing alternatives – individual healing attempt

In addition, the attending physician has a special duty to inform the patient of the possible consequences of off-label use (no manufacturer’s liability etc.)

### Anticonvulsants with effect on neuronal calcium channels

Gabapentin and pregabalin bind with high affinity to the α2-δ-subunit of the voltage-gated calcium channels on peripheral and central nociceptive neurons, thereby reducing the activating calcium influx. A recent Cochrane meta-analysis of the effect of gabapentin [38] could demonstrate a significant pain reduction of > 30% only in PHN and painful diabetic neuropathy. Compared to placebo, gabapentin and pregabalin treatment was associated with more side effects, with severe side effects occurring in only 3%. Side effects included drowsiness, dizziness, edema, gait disturbances, and ataxia.

A further systematic review and meta-analysis [39] showed a combined NNT of 6.3 for gabapentin and 8.3 for delayed release gabapentin or gabapentin-encarbil, with overall good tolerability. There was no evidence of different effects depending on the given dose. The NNT of pregabalin (150–600 mg/day) was 7.7 [39]. A better response was found at a daily dose of 600 mg compared to 300 mg.

The NeuPSIG recommendations [39] strongly recommend the use of gabapentin (in a daily dose of 1200–3600 mg, divided into three doses) and pregabalin (in a daily dose of 300–600 mg, divided into two doses). Gabapentin is approved for the treatment of peripheral neuropathic pain, and pregabalin for the treatment of peripheral and central neuropathic pain. It should be noted that the study situation is not sufficient for all neuropathic pain syndromes.

**Recommendation:** Gabapentin and pregabalin ** shall  be** used as first choice drugs for the treatment of chronic neuropathic pain of any etiology.

### Anticonvulsants with effect on sodium channels

#### Sodium channel blockers: carbamazepine, oxcarbazepine, topiramate

Carbamazepine, oxcarbazepine and topiramate have a membrane-stabilising effect on voltage-gated sodium channels on sensitized nociceptive neurons in the peripheral and central nervous system, thus reducing their spontaneous activity. Topiramate also enhances the inhibitory effect of GABA by binding on GABA_3_ receptors and inhibition of AMPA_2_ receptors.

##### Carbamazepine

A recent systematic review [40] found that due to the lack of available studies, it is not possible to evaluate the evidence of carbamazepine in the treatment of painful diabetic neuropathy. The cross-over studies reporting a positive effect of carbamazepine in the treatment of painful diabetic neuropathy are over 40 years old. A Cochrane analysis [41] and the NeuPSIG recommendations [39] could not provide a valid assessment of the efficacy of carbamazepine in the treatment of neuropathic pain due to the lack of data.

The side effects of carbamazepine are unfavorable [39]. Typical side effects are cognitive disturbances, dizziness, vertigo, fatigue, ataxia, gastrointestinal disorders, hyponatremia, blood count changes (especially leucopenia), liver damage, allergic skin reactions and cardiac arrhythmia [42, 43]. Contraindications are a pre-existing bone marrow lesion, history of allergic reactions to TCAs and simultaneous therapy with an MAO-inhibitor, nefazodone or reverse transcriptase inhibitors. Carbamazepine is an enzyme inductor of the cytochrome P450 system and can therefore interfere with other substances.

**Dosage:** Treatment is started with 100–200 mg carbamazepine (with extended release) and gradually increased to 600–1200 mg under laboratory (blood count, liver values, and electrolytes) and ECG controls. Carbamazepine is in Germany approved for the treatment of trigeminal and glossopharyngeal neuralgia, painful diabetic neuropathy and the treatment of epilepsy.

**Recommendation:** Carbamazepine **cannot be generally recommended** for the treatment of neuropathic pain of any etiology due to the limited evidence, but may be considered in individual cases. The unfavorable side effects, especially hyponatremia, and drug interactions must be taken into account.

For the efficacy in trigeminal neuralgia we refer to the corresponding guideline.

##### Oxcarbazepin

The data regarding the efficacy of oxcarbazepine in neuropathic pain is insufficient. A Cochrane analysis of 2017 found little evidence for the efficacy of oxcarbazepine in painful diabetic neuropathy and radiculopathy with an NNTB of 6 (number needed to treat for an additional beneficial outcome) [44]. A meta-analysis of three RCT found evidence that oxcarbazepine achieves pain reduction compared to placebo in the long-term treatment of painful diabetic neuropathy [42]. However, another meta-analysis of 3 studies showed that the reduction in pain intensity in painful diabetic neuropathy with oxcarbazepine, with a mean value of 5.93 points on a scale of 0–100, is very low compared to other pharmacotherapies [45]. In patients with various types of peripheral neuropathic pain, the administration of oxcarbazepine has been shown to significantly reduce pain severity, especially when there is evidence of preserved nociceptive function in sensitized nociceptors [46]. This could be relevant in painful diabetic neuropathy [47]. An RCT of 55 patients with spinal cord lesions showed that oxcarbazepine is more effective in the absence of stimulus-induced pain [48]. However, the available data is inadequate for final conclusions regarding the pain phenotype [49].

Oxcarbazepine has an unfavorable side effect profile [39]. Typical side effects are drowsiness, dizziness, headaches, nausea, vomiting, cognitive symptoms, hyponatremia, and severe allergic skin reactions.

**Dosage:** The therapy is started with 300 mg/day and increased to a maximum of 1800 mg/day in 2 single doses. The therapeutic dosage is between 1200 and 1800 mg/day. Oxcarbazepine is only approved in Germany for the indication epilepsy, not for pain therapy.

**Recommendation:** Oxcarbazepine **cannot be generally recommended** for the treatment of neuropathy pain of any etiology due to the limited evidence, but may be considered **in individual cases**. The unfavorable side effect profile, especially hyponatremia, must be taken into account.

For the efficacy in trigeminal neuralgia, we refer to the corresponding guideline.

##### Topiramate

No evidence for the efficacy of topiramate at doses of 100–400 mg/day in the treatment of painful diabetic neuropathy could be found in 3 high-quality, larger randomized, double-blind, placebo-controlled studies [50]. A meta-analysis [42] and a current systemic review [40] found no evidence of efficacy of topiramate in painful diabetic neuropathy. For other neuropathic pain, 2 Cochrane analyses also showed no evidence for topiramate [51, 52]. A meta-analysis of 2 studies on pharmacotherapy for painful diabetic neuropathy showed that topiramate, with a mean value of 3.09 point on the VAS from 0 to 100, achieved the lowest pain reduction compared to other anticonvulsants and antidepressants [45]. The side effect profile of topiramte is unfavorable [39]. Typical side effects include cognitive symptoms, fatigue, loss of appetite, and gastrointestinal disorders. Severe skin and mucous membrane reactions and acute eye complications have been reported. Particular attention should be paid to topiramate-induced weight reduction.

**Recommendation:** Topiramate **should not be** used to treat neuropathic pain of any etiology.

### Lamotrigine/lacosamide/phenytoin

#### Lamotrigine

In a Cochrane analysis, lamotrigine was not effective in the treatment of painful diabetic neuropathy [53]. In a further meta-analysis [39] the NNT (for a pain reduction of > 50%) is calculated at 17.8 (9.3–210), the NNH (number needed to harm) at 17.3 (11–44). The effect was observed from a dose of > 200 mg. A slow dosage due to the side effect of an allergic skin (in about 10% [53]) is additionally disadvantageous in clinical application. A small study on central post-stroke neuropathic pain (CPSP) showed a significant pain reduction of about 30% by lamotrigine. A pilot study on HIV neuropathy showed a significant reduction in pain (> 30%), but in a follow-up study this was the case only in the subgroup of patients under antiretroviral therapy. Lamotrigine is in Germany only approved for the treatment of epilepsy and bipolar disorders.

**Recommendation:** Lamotrigine **cannot be generally** recommended in the treatment of neuropathic pain of any etiology, but can be considered as off-label use (especially in HIV neuropathy, central post-stroke pain) in **individual cases**.

For the use in the therapy of trigeminal neuralgia we refer to the corresponding guideline.

#### Lacosamide

There are 3 meta-analyses for the efficacy of lacosamide in painful diabetic neuropathy [39, 52, 54]: In a Cochrane analysis, lacosamide at a dose of 400 mg led to a significant 30% reduction in pain with an NNT of 9.8 (5.7–36, RR: 1.28 CI 1.09–1.49). At the higher dose of 600 mg the NNT was lower, but the rate of side effects was also higher (NNT 4.3 (3.0–7.3, RR 1.8 CI 1.3–2.3) [54]. Another Cochrane analysis, which included 2 studies on diabetic neuropathy, found a significant 50% reduction in pain at a dose of 400 mg lacosamide (NNT 10 (5.2–120, RR: 1.4 CI 1.01–1.9)), but the higher dose of 600 mg failed to demonstrate a 50% reduction in pain [52]. The meta-analysis by Finnerup et al. did not found a significant effect for lacosamide (200, 400 und 600 mg) with respect to a pain reduction of 50% at an NNH of 8.6 (6.3–13; 1314 participants) and therefore does not recommend lacosamide in the therapy of neuropathic pain. In summary, the data is limited to painful diabetic neuropathy with a high NNT for the dose of 400 mg and inconsistent data for lacosamide 600 mg with a high NNT and a similar NNH of 8.6 (6.3–13) [39].

**Recommendation**: Lacosamide **cannot be** recommended for the treatment of neuropathic pain of any etiology, as the available data is insufficient.

#### Phenytoin

For phenytoin, there are no studies of sufficient quality to assess its efficacy [52, 55]. Older, methodologically inadequate studies on efficacy in painful diabetic neuropathy provide contradictory results [56, 57]. Phenytoin is, however, in Germany approved for central or peripheral neuropathic pain, when other treatments have not been successful or are not feasible.

**Recommendation:** Phenytoin **should not** be used in the therapy of chronic neuropathic pain.

For us in acute exacerbation of trigeminal neuralgia, please refer to the corresponding guideline.

### Other anticonvulsants

#### Levetiracetam

In a meta-anaylsis of 7 RCT [39], levetiracetam at a dose of 3000 mg did not reduce pain in different neuropathic pain conditions.

**Recommendation:** Levetiracetam **should not be** used to treat neuropathic pain of any etiology.

### Antidepressants

#### Tri−/tetrazyclic antidepressants

Several meta-analyses and a Cochrane analysis confirm the efficacy of tricyclic antidepressants, but emphasize that the evidence is based on several small studies and is therefore of only moderate quality and that the treatment effect has probably been overestimated [39, 58–60]. Tricyclic antidepressants have no direct antinociceptive properties. They are also effective in patients who do not have depression. The effect on neuropathic pain appears to occur earlier and at lower doses than the effect on depression. Tricyclic antidepressants (amitriptyline, imipramine and clomipramine) bind to norepinephrine and serotonin (5-HT) transporters. The reuptake of these neurotransmitters is inhibited, which activates descending noradrenergic inhibitory pathways [39]. Tricyclic antidepressants also block sodium channels and thus inhibit ectopic discharges.

The number needed to treat (NNT) for a 50% pain reduction is lowest for tricyclic antidepressants. For amitriptyline, imipramine, and clomipramine, which inhibit norepinephrine and serotonin reuptake, a meta-analysis by Finnerup [39] shows that NNT is 2.1, whereas for pure norepinephrine reuptake inhibitors (nortriptyline and desipramine) it is 2.5. However, these studies were conducted some time ago with smaller patient numbers, with a cross-over design and without direct comparison with other substances. There are only 2 studies on imipramine and diabetic neuropathy, so no further statement on individual substances could be made in that meta-analysis. A distinction must be made between sedating (e.g. amitriptyline) and non-sedating (e.g. clomipramine) TCA. Amitripytline can be particularly helpful for insomnia due to neuropathic pain.

**Dosage:** Individual titration is required depending on the effect and side effects. Amitriptyline and imipramine have the advantage of being available as drops. Starting dose: 10–25 mg (extended release) at night or - depending on the active ingredient- also in the morning, dose increase very 3–5 days by 10–25 mg. In elderly patients lower doses should be used, especially at the beginning. Prior to treatment, an ECG should be obtained in all patients at cardiac risk and over 65 years of age. The effective and tolerable dosage is usually between 25 and 75 mg/day (sometimes even lower), depending on the active ingredient, either as a single dose or divided into 2–3 daily doses. Higher doses are only necessary it antidepressive effects are desired (> 150 mg/day). Amitriptyline is in Germany approved for the treatment of neuropathic pain in adults, clomipramine and imipramine for long term pain management as part of an overall therapeutic concept, while trimipramine and nortriptyline are off-label in pain management. Important side effects are: Sedation, dry mouth, cognitive decline, weight gain, constipation, dizziness, orthostatic dysregulation, erectile dysfunction, micturition problems, nausea, tremor and cardiac side effects. Relative contraindications for tricyclic antidepressants include glaucoma, prostate hypertrophy, voiding disorders, an increased risk of seizures, thrombosis, thrombophlebitis, cardiac conduction disorders and heart failure as well as an increased risk of falls. If doses above 100 mg/day are used, regular ECG recordings are recommended, especially for older patients. Laboratory checks of transaminases and blood count before and during therapy are recommended.

The CYP-dependent enzymes lead to a variety of interactions. As an example, amitriptyline must not be combined with MAO-inhibitors (risk of serotonergic syndrome), other anticholinergic (increasing side effects) or adrenergic substances (risk of arrhythmia). Carbamazepine and barbiturates can lower the concentration of tricyclic antidepressants and reduce their effectiveness.

**Recommendation**: Tricyclic antidepressants **shall be** used as fist choice drugs for the treatment of neuropathic pain of any etiology.

In the risk-benefit assessment, however, the side effects, drug interactions and cardiac toxicity of TCA must be taken into account.

### Selective serotonin- and norepinephrine-reuptake inhibitors (SSNRI)

#### Duloxetine

The analgesia is explained by the presynaptic reuptake inhibition of the monoaminergic neurotransmitters serotonin and norepinephrine and thus an amplification of the descending pain-inhibitory pathway. In patients with painful diabetic neuropathy, the SSNRI duloxetine is effective at doses of 60–120 mg per day [61–65]. A dose increase from 60 mg duloxetine per day (single dose) to 120 mg per day (spread over two daily doses) did not show a significantly stronger pain reduction in patients with diabetic neuropathy in a post-hoc analysis [66]. The number needed to treat (NNT) for at least 50% pain reduction after 12 weeks of treatment with duloxetine 60 mg vs. placebo is 5.7 and for duloxetine 120 mg 5.7 [64]. In a randomized, non placebo-controlled head-to-head study [67], there was no difference in analgesic efficacy between amitriptyline, duloxetine and pregabalin in patients with painful diabetic neuropathy. The intake of pregabalin led to an improvement in sleep continuity, that of duloxetine to a shortened sleep duration and an improvement in mobility. The frequency of side effects was increased with duloxetine compared to pregabalin. In daily practice, in the event of ineffectiveness or partial effectiveness, a substance from a different group of active substances should be used. Doses lower than 60 mg duloxetine per day are not effective in the treatment of painful diabetic neuropathy [68].

**Dosage:** It is recommended to start the therapy with a dose of 30 mg in the morning and after 7–14 days increase to the target dose of 60 mg (up to 120 mg) as a single dose in the morning. A maximum dose of 120 mg can be administered. Duloxetine is in Germany approved for the treatment of diabetic neuropathy, depressive disorders and generalized anxiety disorders. All other indications are off-label use.

Serious side effects are rare [68]; nausea and vomiting may occur, especially in the first few weeks. Increases in blood pressure can occur, therefore regular checks are recommended. A worsening of the diabetes may occur. In addition, fatigue, dizziness, increased sweating, dry mouth, constipation, reduced appetite, insomnia, diarrhea, disturbed consciousness and trembling, as well as an increase in intraocular pressure may occur. Before treatment, an ECG should be taken for all patients. Before and during therapy, regular laboratory tests of liver and kidney values and blood count should be performed [69]**.** Contraindications are liver and severe kidney dysfunction and uncontrolled hypertension.

**Interactions:** Duloxetine should not be combined with serotonergically active substances, MAO inhibitors or St. Johns’s wort. CYP1A2 inhibitors (e.g. ciprofloxacin) can lead to an increase in the active level of duloxetine. In smokers, the degradation of duloxetine is accelerated due to CPY1A2 induction, they have lower plasma levels and therefore an increase in dose to 120 mg should be considered. Duloxetine inhibits the breakdown of metoprolol and can therefore double its effective level and increase the risk of bleeding through simultaneous anticoagulation (especially warfarin).

**Recommendation:** Duloxetine **shall be** used as the first choice drug for treatment of neuropathic pain of any etiology.

#### Milnacipran

**Evidence:** There is currently no evidence of a significant effect of the SSNRI milnacipran in neuropathic pain compared to placebo. A Cochrane analysis identified a single study comparing milnacipran 100–200 mg per day with placebo for 6 weeks in patients with chronic back pain radiating to the leg or buttocks and found no evidence of efficacy [70]. In Germany, milnacipran is only approved for the treatment of major depression.

**Recommendation:** Milnacipran **should not be** used to treat neuropathic pain of any etiology.

#### Venlafaxine

**Evidence:** In a Cochrane meta-analysis there were indications that venlafaxine has positive effects on neuropathic pain. However, because some studies had methodological weaknesses and there was a risk of bias [71], the evidence for the efficacy of venlafaxine was insufficient. The effect of venlafaxine in chemotherapy-induced neuropathy was investigated in a randomized, double-blind study [72]. Venlafaxine was administered before the start of chemotherapy and on days 2–11 afterwards. The venlafaxine group achieved significantly more frequent symptom relief and improvement, but the number of patients examined was small and the duration of the study was short. For the treatment of chemotherapy-induced neuropathy, the S3 guideline “Supportive therapy in oncology patients” therefore recommends, according to expert opinion, that this therapy can be considered in the context of chemotherapy-induced neuropathy.

In patients with painful diabetic neuropathy, a double-blind randomized study showed that venlafaxine is more effective than placebo [73]. In a further study with cross-over design, whose methodological quality was poorer and whose case numbers were smaller, patients with painful neuropathy of different etiology were treated with venlafaxine 225 mg/day compared to imipramine 150 mg/day or placebo [74]. The analgesic efficacy of venlafaxine in this study was better than that of placebo and did not differ significantly from imipramine. However, the methodological limitations of the study significantly limit the validity of the effect.

In Germany, venlafaxine is only approved for the treatment of major depression, generalized and social anxiety disorders and panic disorders.

**Recommendation:** venlafaxine **cannot be** recommended for the treatment of neuropathic pain due to insufficient data, but may be considered for off-label use in individual cases.

### Selective serotonin reuptake inhitibors (SSRI)

The efficacy of selective serotonin reuptake inhibitors (SSRI, e.g. fluoxetine, fluvoxamine, citalopram, escitalopram, sertraline) in neuropathic pain has not be proven beyond doubt [39, 75]. The studies conducted have been very small, not randomized and controlled or have shown no relevant effect [76–79].

**Recommendation:** SSRI such as citalopram/escitalopram, fluoxetine, fluvoxamine or sertraline **should not be** used in the treatment of neuropathic pain.

### Noradrenergic and specifically serotonergic antidepressants (NaSSA)

The efficacy of noradrenergic and specifically serotonergic antidepressants (NaSSA; z. B. mirtazapine) in neuropathc pain could not be proven beyond doubt, although these substances have the advantage over SSRIs af additionally influencing the noradrenergic transmitter system. A combination of mirtazapine and low-dose pregabalin has been shown to be helpful in the treatment of painful bone metastases with neuropathic pain component compared to monotherapy with pregabalin, although the study involving 37 patients was very small [80]. However, further studies with larger patient cohorts are lacking.

**Recommendation:** NaSSA **should not** be used for neuropathic pain of any etiology.

### Opioids

Opioids act as agonists mainly at the μ-opioid receptor in the central nervous system. Depending on the intrinsic activity at the receptor, a distinction is made between low-potency (weak) and high potency (strong) opioids. In addition, there are substances which, in addition to their action at the μ-receptor, act on the descending pain inhibitory system via noradrenergic and serotonergic reuptake inhibition.

In a consensus statement of the Canadian Pain Society [81], opioids are recommended as a second-choice treatment for chronic neuropathic pain. In contrast, a review article by Finnerup et al. [39] recommends weak opioids as second-line treatment, while strong opioids are only recommended as third-line medication. This graduation is justified by the potential for abuse of strong opioids in chronic use and the increasing mortality due to overdose. A meta-analysis by Sommer et al. [82], showed that opioids in therapeutic use for chronic neuropathic pain (duration of the study was only 12 weeks) were superior to placebo in terms of efficacy, but inferior in terms of tolerability.

**Tramadol** is an opiate agonist with weak affinity to the μ-opioid receptor. The relative analgesic potency in relation to morphine is stated as 0.1. Tramadol also acts as a serotonin and norepinephrine reuptake inhibitor. This mechanism of action provides an additional analgesic and pain modulating effect via descending inhibiting pathways in the spinal cord. In a meta-analysis [83], tramadol showed significant pain relief, although the evidence was limited due to the small number of participants.

**Tapentadol** also has a dual mechanism of action as a μ opioid receptor agonist (MPR) and norepinephrine reuptake inhibitor. So far, however, the evidence for the evaluation of tapentadol in the treatment of neuropathic pain is insufficient due to the limited data available [84, 85].

Cooper et al. [86] found in a Cochrane review insufficient evidence for the efficacy of **morphine and oxycodone** in the treatment of various neuropathic pain conditions, NNT 4.3 (95% CI 3.4–5.8) and NNH 11.7 (8.4–19.3) combined for oxycodone (10–120 mg/day) and morphine (90–240 mg/day). The maximum effect was shown at doses of 180 mg morphine or morphine equivalent without additional benefit at higher doses.

For **oxycodone,** a Cochrane review [87] showed an improved outcome (NNTB 5.7) in diabetic neuropathy or postherpetic neuralgia, but the evidence was not considered sufficient for recommendation.

In a systematic review concerning the importance of **hydromorphone** in the treatment of neuropathic pain [88], only one post-hoc analysis out of 4 RCTs could be included with a positive effect. On the basis of the data available, however, the efficacy of hydromorphone cannot be assessed with certainty.

In a systematic review of **buprenorphine** [89], none of 11 published studies met the inclusion criteria, therefore no statement could be made concerning the efficacy of buprenorphine in neuropathic pain.

In a systematic Cochrane review of **methadone** [90], only 3 studies could be included, but their low methodological quality did not allow any statement about the efficacy of methadone in the treatment of neuropathic pain.

For the synthetic opioid **fentanyl** only one study on transdermally applied fentanyl was identified in a systematic review [91]. Due to the low number of participants and the high number of dropouts, the quality of the study is considered low and the results cannot be assessed with certainty.

Opioids have been shown to be more effective than placebo in diabetic neuropathy and post-herpetic neuralgia, and positive data are also available for post-amputation pain, back pain and spinal cord injury pain. Dosage recommendations are only available for morphine. However, there is no convincing evidence that morphine at doses of up to 180 mg/day is effective in the long-term treatment of neuropathic pain (at least 12 weeks and longer). However, this does not exclude the possibility that certain groups of patients may benefit from treatment with morphine. There is limited evidence for therapy with oxycodone, but a moderate benefit is seen in diabetic neuropathy or post herpetic neuralgia. There are too few studies available for hydromorphone to date to assess an effect on neuropathic pain. Weak opioids such as tramadol cause fewer undesirable side effects such as constipation or dizziness, but overall the evidence is insufficient. For buprenorphine, methadone and fentanyl, there is insufficient evidence for neuropathic pain. Due to adverse side effects, treatment with fentanyl led to premature termination of the study in half of the study participants.

**Dosage:** In short-term treatment doses of morphine above 180 mg/day or equipotential doses of other opioids should not be exceeded. In the case of treatment over longer periods of time, the spectrum of side effects (especially somnolence, sedation, constipation, and nausea) and the development of tolerance limit the analgesic benefit.

**Recommendation:** Low potency μ opioid receptor agonists and norephrine reuptake inhibitors such as tramadol as well as high potentcy opioids **can be** used as a third line treatment of neuropathic pain of any etiology. Undesirable side effects, development of tolerance and comorbid additions can limit the application.

### Cannabinoids

Cannabinoids are agonists at CB1 receptors in the CNS, spinal cord and peripheral nerves. They act by inhibiting neuronal excitability [92]. A meta-analysis of 11 studies found a significant, but clinically only small pain reduction by cannabinoids. In another meta-analysis [93] cannabinoids significantly reduced neuropathic pain, although the individual data were heterogeneous. Cannabis smoked or absorbed through mucous membranes seemed to be more effective than cannabis administered orally. In the meta-analysis by Petzke et al. [94] cannabinoids also showed a significant reduction in pain, but this effect was not clinically relevant with an NNTB of 14, while the central nervous and psychiatric side effects were significantly and relevantly more frequent with an NNTH (number needed to treat for an additional harm) of 3 and 8 respectively. A meta-analysis on the use of inhaled cannabis found a significantly more frequent 30% pain relief among cannabinoids compared to placebo with an NNTB of 5.6; however, only very short term effects (between 5 h and a maximum of 2 weeks) were investigated and only 5 RCTs were included. The meta-analysis by Iskedjian et al. [95] also showed a significant, but only slightly pronounced effect of cannabinoids on pain intensity, which was no longer significant after correction for 2 studies that had allowed many other analgesics. A review by Hauser [96] summarized several meta-analyses and concluded that the data on the efficacy of cannabinoids are inconsistent. Overall, only few data on the long-term effect and long-term safety were available. In the NeuPSIG recommendations [39], a weak recommendation is given against the use of cannabinoids, as only 2 of 9 studies examined had a positive effect, using the criterion of 50% pain reduction. According to the recommendations of the National Guidelines for Care in diabetic neuropathy (Nationale Versorgungsleitlinie), cannabinoids should not be used in painful diabetic neuropathy. In a recent Cochrane review, the 30% pain reduction was more frequent with cannabinoid use, but the effect was rather small with an NNTB of 11 and significantly more central side effects occurred with an NNTH of 3, so that the small effect of cannabinoids may be antagonized by the side effects [97]. In an EFIC (European Pain Federation) position paper, cannabinoids should only be considered for neuropathic pain after failure of standard therapies in a multimodal setting [98].

Overall, several meta-analyses showed a reduction of neuropathic pain, but this effect was rather small and the therapy led significantly more often to central and psychiatric side effects.

In 2017 a new law came into force in Germany (“Cannabisgesetz, Cannabis Act”), which made cannabinoids (cannabis flowers, cannabis extracts, dronabinol, nabilone, nabiximols) reimbursable upon application to health insurance funds. The patient must submit a case-by-case application for the costs to be covered by the statutory health insurance. After approval, these substances can be prescribed by means of narcotic (BTM) prescription. It is still an off-label use, as none of these substances is approved for the indication “pain”. The “Cannabis Act” obliges the prescribing physician to conduct an accompanying survey to record the success of the therapy.

**Dosage:** Available are dronabinol (semi-synthetically produced tetrahydrocannabinol (THC), dosage 2.5–10 mg/day), nabilone (fully synthetically produced THC, dosage 1–4 mg/day) and a combination of THC and cannabidiol (CBD, nabiximols) as an oromucosal spray [94]. In addition, cannabis flowers and cannabis extracts can be prescribed, but note that the THC content varies depending on the flower variety and origin. Currently, only the combination (THC/CBD (nabiximols)) is approved as a nasal spray for the treatment of spasticity in multiple sclerosis (MS) at a dosage of 1–12 strokes/d (corresponding 2.7–32.4 mg THC/2.5–30 mg CBD).

**Recommendation**: Cannabionoids **cannot be** recommended for the treatment of neuropathic pain of any etiology, because the effect is rather small and the rate of side effects is high. Only in individual cases, in the event of failure of other pain therapies, can the use of cannabinoids as off-label therapy be considered within a multimodal therapy concept.

#### Alpha lipoid acid

Alpha lipoic acid is a radical scavenger. All studies have only been conducted in patients with diabetic neuropathy. A meta-analysis of 6 RCT showed a significant improvement in the total sum score (TSS), especially after i.v. administration [99]. A meta-analysis using 4 RCT also found a significant improvement in TSS; however, this effect was unlikely to be clinically relevant for oral administration with less than 30% improvement in TSS, while the effect was greater for i.v. administration (2 studies), so that this was classified as potentially effective. However, long-term data are not available [100]. A meta analysis from 2004 [101] found a significant 50% response rate on the improvement in TSS. However, none of the included studies reported the amount of pain reduction or a 30% pain reduction, as only one cumulative score, TSS, was used, which is not very common in other studies [102]. The most recent meta-analysis with 5 RCTs found a significant effect on the pain subscore of TSS, but due to the short duration of the studies and a high risk of bias, the evidence level was assessed as low [102]. A long-term study using 600 mg alpha lipoic acid daily vs. placebo for 4 years showed no effect on the primary endpoint (improvement in “composite score” consisting of neuropathy impairment score as a measure of negative symptoms and 7 neurophysiological tests) and the TSS; only the neuropathy impairment score itself was significantly improved and the therapy was well tolerated [103].

**Dosage:** 600 mg alpha lipoic acid per day. In Germany the preparation is not reimbursable, but approved for the treatment of paresthesias in diabetic neuropathy.

**Recommendation:** Alpha lipoic acid **cannot be** recommended for the treatment of neuropathic pain of any etiology. An effect in diabetic neuropathy cannot be excluded. However, the evidence is not sufficient to generally recommend its use in diabetic neuropathy.

### NMDA receptor antagonists

The effect is achieved by inhibition of the N-methyl-d-aspartate (NMDA) receptor and thus reduction of glutamate release, e.g. in the posterior horn of the spinal cord, but also in other parts of the CNS (Central nervous system). In a review article, different NMDA receptor antagonists were examined: Ketamine had a relevant analgesic effect in the intravenous application, memantine had no effect, methadone showed only a slight analgesic effect in 3 of 3 studies; amantadine had a slight effect in 2 of 3 studies, but only when applied intravenously. Overall, the studies were small and a meta-analysis was not feasible due to the heterogeneity of the data, which clearly limits the significance of the results [104]. In a review of 12 studies with different oral NMDA receptor antagonists (memantine, magnesium and dextromethorphan) only 2 were positive. Overall the NNT was given as 5.0 (3.6–8.1) and the NNH as 9.4 (6.2–25); however, with inconsistent data, it was not possible to issue a NeuPSIG recommendation [39]. A small, uncontrolled study involving 32 patients showed an effect of ketamine in chronic neuropathic pain with an unacceptable high rate of side effects in both intravenous and oral therapy [105]. In another randomized study, intravenously administered ketamine was also effective, but with unacceptable side effects [106].

**Recommendation:** NMDA receptor antagonists **should not** be used to treat chronic neuropathic pain of any etiology.

### Non-opiod analgesics

Although 40% of patients with neuropathic pain take NSAIDs (non steroid analgesic anti-inflammatory drugs), there is little data on the efficacy of these drugs [107]. A Cochrane analysis on the use of NSAIDs in neuropathic pain could only include 2 studies of patients with back pain with a neuropathic component and PHN [108]. These data could not demonstrate a significant pain reduction by NSAIDs. Not a single study could be included in a Cochrane analysis of the use of paracetamol (acetaminophen) in neuropathic pain [109] and no relevant study could be found for metamizole. These drugs can have dangerous side effects such as kidney damage and bleeding, especially gastrointestinal bleeding, when used over a long period of time. Paracetamol can be hepatotoxic in high doses and metamizole has a risk of agranulozytosis, although rare.

**Recommendation:** Non-opioid analgesics (NSAIDs, Cox-2 inhibitors, paracetamol, metamizole) **should not** be used for the treatment of chronic neuropathic pain of any etiology, as there is no evidence of efficacy.

### Muscle relaxants

#### Baclofen

Baclofen is a specific agonist at the GABA_B_ receptor. Baclofen is approved for the treatment of spasticity. There are several studies on baclofen for the treatment of trigeminal neuralgia (for details see the guideline “trigeminal neuralgia” of the DGN), but no randomized placebo-controlled trials for the treatment of other neuropathic pain states, only some open studies.

**Recommendation:** Baclofen **should not** be used to treat neuropathic pain of any etiology.

#### Tizanidine and tolperisone

No studies could be found for these preparations, so that tizanidine and tolperisone will not be discussed further.

#### Flupirtine

The Pharmacovigilance Committee (PRAC) of the European Medicines Agency recommended 2018 to revoke the approval due to its hepatotoxic potential and the suppliers withdrew their preparations from the market in the EU (European Union). Therefore it is not discussed any further.

### Benzodiazepines

Benzodiazepines are agonists at inhibitory GABA receptors in the CNS and act by inhibiting neuronal excitability. In a meta-analysis no controlled studies that met the requirements could be found [110].

**Recommendation:** Benzodiazepines **should not** be used to treat neuropathic pain of any etiology.

### Topical therapies

#### Lidocaine patch

Lidocaine prevents the development of ectopic action potentials by blocking voltage-dependent sodium channels. In addition, a reduction in epidermal nerve fiber density has been described with prolonged use. The effectiveness of lidocaine patches (5%) in post-herpetic neuralgia (PHN) has been demonstrated in several studies. Besides the local anesthetic effect, it offers protection against mechanical stimulation (mechanical dynamic allodynia), which is a common problem in PHN).

In patients with painful diabetic neuropathy, an open randomized trial showed an effect comparable to pregabalin after 4 weeks [111]. However, a Cochrane-review could not provide sufficient evidence due to the poor quality of the randomized trials [112]. There are several reports of a positive effect and the spectrum of side effect is very small, therefore its use is recommended in many guidelines [39].

**Dosage:** 1–3 patches (700 mg/patch, 10 × 14 cm) are applied to dry, intact, non-irritated skin in the painful area for 12 h. Between patches an application free interval of at least 12 h must be observed. The patches can be cut to size so that smaller areas can also be treated. A maximum of 3 patches can be applied every 24 h hours. Approval exists only for PHN; all other indications are off-label use.

Possible side effects are local skin reactions such as erythema, itching and very rarely blistering. Due to the low systemic absorption rate, no central side effects or interactions are expected, which is particularly beneficial in older patients [39]. Overall, treatment with lidocaine patch can be considered safe [113]. A tolerance development has not been described. Contraindications are intolerances and open skin wounds. The application site should be inspected as part of the follow-up examinations, in case of local skin reaction, the application area should be changed or a therapy break should be taken.

**Recommendation:** Lidocaine patch **can be** recommended as second line treatment of localized neuropathic pain. Its efficacy has been shown in particular in PHN. In PHN the primary used should also be considered.

#### Capsaicin

Capsaicin is the active ingredient of chili pepper and acts as a natural ligand of TRPV1 receptor (TRPV1; transient receptor potential cation channel subfamily V member 1). In a Cochrane review of 8 RCT in 2488 patients, the majority of patients showed a moderate to significant pain relief of the capsaicin 8% patch compared to placebo or patches containing only 0.04% capsaicin. However, the quality of the evidence was moderate to very low and the proportion of the patients who benefited from treatment was small. The effect of the capsaicin 8% patch was found to be similar to other treatment options for neuropathic pain [114]. In a systematic review and meta-analysis of the treatment of neuropathic pain which examined 229 studies a weak recommendation as a second choice drug war made [39]. In an early meta-analysis of the Qutenza Clinical Trials Database including 1458 patients in 7 studies, the authors conclude that capsaicin 8% is superior to a lower dose in patients with painful HIV neuropathy and PHN [115]. In another systematic review of 25 studies with painful diabetic neuropathy, the capsaicin 8% patch was superior to placebo, better than pregabalin and gabapentin, and similar to duloxetine [116]. Overall, a reduction in neuropathic pain was shown in several meta-analyses. This effect was comparable to effects of oral medications for neuropathic pain with fewer side effects.

**Dosage:** Capsaicin 8% patch is available in Germany as a formulation containing 179 mg capsaicin. It is applied to the painful area of the body for a maximum of 60 min, with a maximum of 4 patches applied simultaneously. The patches can be cut to a smaller size. The treatment can be repeated every 90 days. Pre-treatment with lidocaine cream or oral analgesics can be performed. The patch is in Germany approved for the treatment of peripheral neuropathic pain in adults.

**Recommendation**: Capsaicin 8% patch **can be** recommended for the treatment of neuropathic pain of any etiology as second choice. The effect is comparable to that of established oral medications with good tolerability. Primary use should be considered for localized neuropathic pain.

### Botulinumtoxin

Botulinum toxin (BTX) acts at the neuromuscular junction and blocks the release of acetylcholine from its presynaptic vesicles by cleaving the SNARE proteins (soluble N-ethylmaleimide), resulting in muscle paralysis. However, the effect on neuropathic pain appears to be independent of the effect on muscles [117] and seems to be mediated by a reduction of the release of pro-inflammatory mediators from peripheral nerves and dorsal root ganglia (substance P, CGRP (calcitonin gene-related peptide), glutamate).

A meta-analysis of 2 small studies with only 58 patients on diabetic neuropathy found a significant pain reduction without significant side effects [118]. A further meta-analysis including 6 RCT on PHN and trigeminal neuralgia [119], found a significant reduction in pain and a significant rate of patients with 50% pain relief, however, with risk of bias. In NeuPSIG recommendations [39], NNT is calculated with 1.9 (4 RCT), but with one additional negative study, so that a weak recommendation is made for the use of BTX. A larger RCT in 66 patients with post-traumatic neuralgia, painful neuropathy or PHN found a significant pain reduction (NNT 2.5) [120].

In summary, the data suggest that BTX is effective with a demonstrable 30% pain reduction, but the number of treated patients in the trials has been very small and in some cases a risk of bias has been described.

**Dosage:** 50–200 units botulinum toxin A (onabutulinumtoxin A). BTX is not approved for this indication.

**Recommendation:** Botulinum toxin **can be considered** for the treatment of neuropathic pain of any etiology, but only as a third choice drug for focal limited pain in specialized centers.

### Amitriptyline ointment

In a systematic review of 5 RCT and 2 uncontrolled studies no evidence of the efficacy of topically applied amitriptyline could be found, whereas positive effects were only reported by individual case reports and therefore showed a high risk of bias [121]. In a small randomized placebo-controlled study, the topical application of amitriptyline had no analgesic effect in contrast to lidocaine [122].

**Recommendation**: The topical application of amitriptyline ointment **should not** be used for the treatment of chronic neuropathic pain of any etiology.

### Transcutaneous electrical nerve stimulation (TENS)

The mode of action of TENS is controversially discussed in the literature. The effect depends on the stimulus frequency. Low frequency stimulation (1–10 Hz) excites especially A- and A-delta fibers and high frequency stimulation (about 80–150 Hz) rather A beta fibers. This leads to a modulation of the spinal nociceptive transmission. Low-frequency stimulation induces the release of dynorphin. In addition to these spinal effects, the descending pain modulation is probably also activated by TENS (especially by low frequency stimulation).

In TENS application, peripheral nerves are electrically stimulated via skin electrodes. The electrical impulses of the various battery-powered stimulation devices are variable in stimulus form, amplitude, impulse duration and frequency. Stimulation is either performed directly above the pain area or above the main nerve trunk that innervates the pain area. The stimulus induced paresthesias should always cover the pain area. Sometimes, stimulation is also effective contralateral to the pain area. There are only few controlled studies. In a recent Cochrane meta-analysis, it is stated that the quality of the available studies on the effectiveness of TENS in neuropathic pain is low [123]. In conclusion, an analgesic effect of active TENS was found in comparison to a sham-stimulation. However, from the perspective of evidence-based medicine, it was not possible to make a reliable statement regarding efficacy [123].

**Therapeutic approach:** Despite long experience with TENS, its success in individual cases cannot be predicted. Correspondingly, test stimulation is necessary prior to prescription. A pain reduction is reported in up to 60% of all patients with various pain syndromes. However, it should be avoided to stick the electrodes directly above areas with allodynia. In PHN, TENS helps especially in patients with preserved sensitivity. Even in central pain, occasional effects of TENS were observed. In most cases TENS is applied 2–4 times a day for about 30 min.

**Recommendation**: TENS (transcutaneous electrical nerve stimulation) **cannot be** recommended for the treatment of neuropathic pain of any etiology due to the lack of evidence. Since some studies suggest, that it might be effective, its use may be considered in individual cases.

### Various therapies

The intravenous administration of lidocaine and the topical application of ambroxol, clonidine, ketamine and acetylsalicylic acid is not discussed due to the lack of data.

#### Psychotherapeutic interventions

Like other chronic pain disorders, neuropathic pain can only be understood considering the “bio-psycho-social pain model”. Although there are no RCT studies on specific psychological risk factors for development and chronification for most of the neuropathic pain conditions, there is clinical consensus that neuropathic pain is associated with psychological symptoms (depression, anxiety, impulse control disorders etc.) to varying degrees. Pain psychotherapy is therefore an important and a central component of a multidisciplinary therapy concept. Psychotherapeutic interventions are usually of crucial importance for the overall success of pain management, as they can also contribute to improved acceptance, compliance and quality of life of patients.

A Cochrane analysis [124] found only 2 studies with a total of 105 participants that fulfilled the inclusion criteria: In a multicenter RCT study, the short and long-term effects of cognitive behavioral therapy were compared to a waiting control group with 61 patients with pain after spinal cord injury (SCI) [125]. The other RCT [126] tested the efficacy of psychotherapeutic group intervention in 24 patients with burning mouth syndrome compared to 20 patients who received placebo medication only. Both studies could not provide sufficient evidence for the explicit benefit of psychotherapeutic treatment compared, but were also subject to a high bias.

**Recommendation:** Psychotherapeutic treatment approaches **can be** used in the treatment of neuropathic pain of any etiology. So far, however, a 30% reduction of pain could not be proven due to the insufficient data available. Nevertheless, pain psychotherapy represents an important therapeutic option, especially in the context of interdisciplinary multimodal treatment.

#### Multimodal pain therapy

The basis for multimodal pain therapy is a “bio-psycho-social model” of pain development [127]. In addition to physiotherapy and occupational therapy, psychological support is also important. However, the data regarding multimodal pain therapy for neuropathic pain is very limited. A small but uncontrolled study showed a long-lasting positive effect of multidisciplinary cognitive behavioral therapy [128]., while another study found no clear pain reduction, but an improvement in coping strategies in an interdisciplinary pain program [129].

**Recommendation:** The available data regarding a 30% pain reduction for the use of multimodal pain therapy is not sufficient to be able to derive a general recommendation. Nevertheless, multimodal pain therapy is an important therapeutic option for chronic neuropathic pain that is difficult to treat.

## Data Availability

The original and complete version of the guideline and the guideline report are available in German via the homepage of the DGN (Deutsche Gesellschaft für Neurologie).
